# Study on the Interaction Effect of Heavy Metal Cadmium in Soil–Plant System Controlled by Biochar and Nano-Zero-Valent Iron

**DOI:** 10.3390/ijms26094373

**Published:** 2025-05-04

**Authors:** Jiarui Wang, Rangzhuoma Cai, Zhaozhao Hu, Liqun Cai, Jun Wu

**Affiliations:** 1 College of Resources and Environmental Sciences, Gansu Agricultural University, Lanzhou 730070, China; wangjr0503@163.com (J.W.); penguin.hzz@gmail.com (Z.H.); 2 Key Laboratory of Dry Land Crop Science, Gansu Agricultural University, Lanzhou 730070, China; 18394795583@163.com; 3 College of Forestry, Gansu Agricultural University, Lanzhou 730070, China

**Keywords:** cadmium toxicity, biochar, nano zero-valent iron, pakchoi, plant physiology, soil amendment

## Abstract

The accumulation of heavy metal cadmium (Cd) in farmland soil in edible parts of crops seriously threatens plant growth, human health, and even the global ecological environment. Finding stabilization remediation technology is an important means to treat Cd-contaminated soil. This study comprehensively evaluated the synergistic effects of independent or combined application of biochar (BC) (10, 30 g kg^−1^) and nano zero-valent iron (nZVI) (0.1% *w*/*w*) on soil properties and morphological and physiological traits of pakchoi (*Brassica rapa* L. subsp. *chinensis*) under Cd (1, 3 mg kg^−1^) stress by pot experiments. It was shown that Cd toxicity negatively affected soil properties, reduced pakchoi biomass and total chlorophyll content, and increased oxidative stress levels. On the contrary, the combined application of BC (30 g kg^−1^) and nZVI (0.1%, *w*/*w*) reduced the Cd accumulation in the shoot parts of pakchoi from 0.78 mg·kg^−1^ to 0.11 mg·kg^−1^, which was lower than the Cd limit standard of leafy vegetables (0.20 mg kg^−1^) in GB 2762-2017 “National Food Safety Standard”. Compared with the control, the treatment group achieved a 61.66% increase in biomass and a 105.56% increase in total chlorophyll content. At the same time, the activities of catalase (CAT) and superoxide dismutase (SOD) increased by 34.86% and 44.57%, respectively, and the content of malondialdehyde (MDA) decreased by 71.27%. In addition, the application of BC alone (30 g·kg^−1^) increased the soil pH value by 0.43 units and the organic carbon (SOC) content by 37.82%. Overall, the synergistic effect of BC (30 g kg^−1^) and nZVI (0.1% *w*/*w*) helped to restore soil homeostasis and inhibit the biotoxicity of Cd, which provided a new option for soil heavy metal remediation and crop toxicity mitigation.

## 1. Introduction

In recent years, due to the superposition and continuous input of various non-point source pollutions such as mining, metal mining and smelting, industrial traffic emissions, and agricultural chemicals dosing, more than 16.76% of cultivated land is polluted by heavy metals [1]. In these contaminated environments, cadmium (Cd) is a toxic heavy metal that lacks any known beneficial role in plant metabolism [2], its high mobility and biotoxicity pose a serious threat to plant growth and human health [3]. Compared with other toxic elements, Cd is more easily enriched in leafy vegetables and causes oxidative stress by inducing active oxygen species, destroying DNA, inhibiting root metabolism and leaf photosynthesis, thus interfering with plant growth and development, and finally affecting the ecological environment and human health through food chain pathway [4]. The accumulation of Cd in crops is controlled by its bioavailability in soil, which in turn affects the interaction between plants, soil, and the environment [2]. Therefore, studying the mechanism of Cd migration in soil–plant systems and treating Cd-contaminated soil has become a major environmental problem that urgently needs to be solved in China.

Plants themselves have evolved several strategies to establish defense mechanisms in the face of Cd stress [5,6,7,8]. However, these tolerance mechanisms lose their effect at higher concentrations of stress, causing the plant to show symptoms of toxicity [9,10]. Therefore, rational actions to mitigate phytotoxicity are imminent and the search for sustainable and stabilizing remediation technologies is an important tool for the management of Cd-contaminated soils. Biochar (BC) is a class of highly aromatic and highly stable carbon-based materials formed by slow, high-temperature thermal cracking of biological organic residues under anaerobic or anoxic conditions [8,11]. The substance has a stable carbon matrix, large specific surface area, and high porosity, which can immobilize and adsorb trace metals while reducing their bioavailability, making it a promising adsorbent for the removal of pollutants from soils and water bodies [12,13]. Numerous studies have shown that BC not only promotes metal stabilization in soils through processes such as adsorption, metal exchange, and surface complexation [14,15,16] but it has also been shown to increase the organic matter content of the soil and enhance soil functioning [17], regulating microbial community structure and protecting soil homeostasis [18,19]. Therefore, biochar is commonly used in Cd soil remediation to reduce the bioavailability of Cd and plant antioxidant response.

However, due to the increasing Cd pollution year by year in the actual environment, the method of fixing pollutants using biochar alone has been severely challenged [20,21,22]. Adding biochar to the soil reduces the bioavailable fraction of Cd while at the same time increasing the persistence of this pollutant in the soil by impeding the natural process of biodegradation [23]. When biochar enters the soil, it undergoes various aging processes [24,25], chemical changes on its surface and changes in its charge characteristics make the biochar structure less stable [26]. In addition, the surface of the biochar is oxidized, which not only reduces the adsorption capacity of the pollutants, but can even lead directly to the release of already adsorbed pollutants [25]. Therefore, to maintain the sustainability of remediation, domestic and foreign scholars began to turn their attention to the combined remediation technology system, that is, a solution that can be based on the remediation method of “abundant” biochar, which provides new ideas for remediating heavy metal contaminated soil–plant systems [27,28,29].

Several studies have shown that nano zero-valent iron (nZVI) can also be used to remove heavy metals from soil [30,31]. Iron nanoparticles are ultrafine iron particles with a diameter of 1 to 100 nm [32], in the field of environmental remediation, the surface effect, the quantum effect, the small size effect, and the controllable risk of aging products have shown great potential [33,34]. The high reactivity of nZVI is related to its unique core–shell structure, in which Fe^0^ within the nucleus is extremely reductive and can provide electrons to reduce heavy metals [35]. At the same time, by the corrosive effect of air and water, the OH^−^ concentration on the surface rises so that heavy metal ions are precipitated, and the dissolved Fe^2+^ and Fe^3+^ can continue to co-precipitate with the oxygen-containing anions in the aqueous solution, generating a variety of iron (hydrogen) oxides, and improving the shear strength of the soil [36]. Regarding the above problems of biochar, its co-application with nano zero-valent iron is an effective solution. The combined use of the two achieves the synergistic improvement of dispersion and specific surface area, as well as the dual guarantee of aging stability. At the same time, in addition to achieving the main goal of BC with nZVI, i.e., removing potential contaminants from the soil, it is also important to limit the spread of nZVI in the medium and keep it in the contaminated environment, a function that can be fulfilled by BC. The existing studies on the combined application of BC and nZVI mainly focus on the surface modification technology of BC by nZVI [37,38], but this process has limitations such as high cost, time consumption and requirement of special preparation equipment. This study presents a simple and effective solution, namely physical mixing of BC and nZVI with contaminated soil. It is worth noting that the heterogeneity of soil environment will directly lead to differentiated activity responses of BC and nZVI under individual and combined application conditions [39].

Current studies mostly focus on pollution control in a single medium (water or soil) [40,41], while the regulation mechanism of the combined application of BC and nZVI in soil–plant systems has not been fully analyzed. In fact, the application of soil amendment aims to achieve the synergistic goal of soil remediation and ecological function improvement, which has dual value for building a green management mode of “remediation and production” of polluted farmland. In this context, this study comprehensively evaluated the effects of biochar (BC) and nano-zero-valent iron (nZVI) alone and combined application on soil physical and chemical properties, cadmium (Cd) bioavailability, and the morphology and physiological metabolism of pakchoi under Cd stress through pot experiments. At the same time, the mechanism of combined application of BC and nZVI on Cd enrichment, migration, and transformation in the soil–pakchoi system was preliminarily explored. This study provides an innovative application path for the simultaneous remediation and safe production of heavy metal contaminated soil.

## 2. Results

### 2.1. Effect of Biochar and Nano Zero-Valent Iron on Soil pH and SOC

The effects of different treatments on soil pH and SOC content are shown in Table 1. Adding BC can significantly increase soil pH and SOC content (*p* ≤ 0.05), while applying nZVI has little effect on them, and the difference between treatments is not significant (*p* > 0.05). Compared with the B0 treatment, soil pH and SOC content increased with the increase in BC addition, and the difference between treatments reached a significant level (*p* ≤ 0.05). When the soil Cd content was 0 mg kg^−1^, the soil pH value of the B30 treatment increased significantly by 0.42 units, and the SOC increased by 41.17% compared with the control. When the soil Cd content was 1 mg kg^−1^, the soil pH value of the B30 treatment increased significantly by 0.40 units, and the SOC increased by 36.90% compared with the control. Under the condition of Cd3, the soil pH of B30 treatment significantly increased by 0.42 units, and the SOC increased by 37.82% compared to the control.

### 2.2. Effect of Biochar and Nano Zero-Valent Iron on Different Forms of Cd Content in Soil

**Figure 1:**
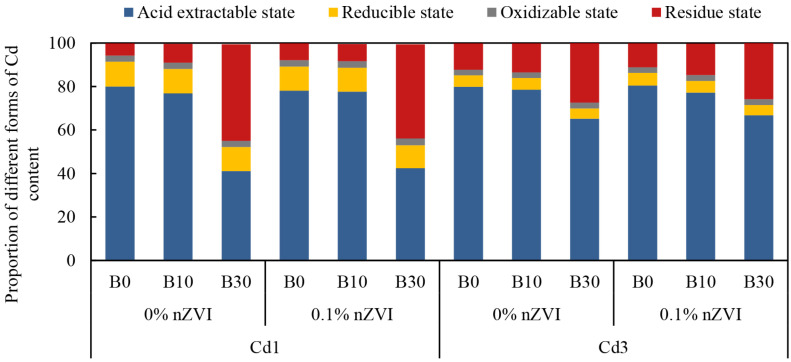
Effect of biochar and nano zero-valent iron on soil Cd morphology in Cd contaminated soil. B = Biochar; nZVI = nano zero-valent iron; Cd1 = 1 mg kg^−1^; Cd3 = 3 mg kg^−1^; 0% nZVI = 0% *w*/*w* nZVI; 0.1% nZVI = 0.1% *w*/*w* nZVI; B0 = 0 g kg^−1^; B10 = 10 g kg^−1^; B30 = 30 g kg^−1^.

As can be seen from Figure 1, the different treatments had different distributions of different forms of Cd content in the soil, and the addition of BC could significantly increase the Cd content in the residue state, and at the same time decrease the Cd content in the acid-extractable state, while the addition of nZVI did not have a significant effect on the content of Cd in the different forms. Under the experimental conditions, the distribution ratio of Cd species in soil was as follows: acid extractable Cd > residual Cd > reducible Cd > oxidizable Cd, acid extractable Cd existed more in soil. Under the Cd1 condition, compared with B0 treatment, the distribution ratio of acid extractable Cd in B30 treatment decreased by 38.96%, the reducible Cd decreased by 0.27%, the proportion of oxidizable Cd increased, but the difference was not significant, and the residual Cd increased by 38.83%. Under the condition of Cd3, acid-extractable Cd in B30 treatment decreased by 14.68% compared with the control, and reducible Cd decreased by 0.53% compared with the control, the proportion of oxidized Cd increased, but the difference was not significant, the residual Cd increased by 15.18% compared with the control. Compared with the control, there was no significant difference in the distribution of different forms of Cd in soil treated with nZVI, but with the increase in soil Cd content, the distribution ratio of acid-extractable Cd in soil increased significantly, at the same time, with the increase in BC dosage, its distribution ratio decreased, but the decrease was less than that of low Cd stress.

### 2.3. Effect of Biochar and Nano Zero-Valent Iron on Biomass and Physiological Metabolism of Pakchoi

#### 2.3.1. Effect of Biochar and Nano Zero-Valent Iron on Pakchoi Biomass

**Figure 2:**
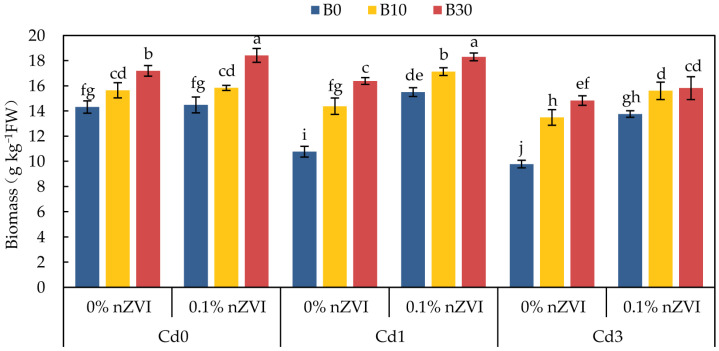
Effect of different treatments on pakchoi biomass. Data are shown as mean ± SD (n = 3). This means sharing the same letters, for a parameter, does not differ significantly at *p* ≤ 0.05. B = Biochar; nZVI = nano zero-valent iron; Cd0 = 0 mg kg^−1^; Cd1 = 1 mg kg^−1^; Cd3 = 3 mg kg^−1^; 0% nZVI = 0% *w*/*w* nZVI; 0.1% nZVI = 0.1% *w*/*w* nZVI; B0 = 0 g kg^−1^; B10 = 10 g kg^−1^; B30 = 30 g kg^−1^.

It can be seen from Figure 2 that the addition of BC and nZVI alone and the combined application of both can significantly increase the biomass of pakchoi and promote the growth of pakchoi. Compared with no Cd stress, the biomass of pakchoi polluted by Cd was significantly affected, which decreased by 24.81% and 31.66%, respectively. Under low Cd stress, the effect of BC and nZVI combined treatment on pakchoi yield was higher than that of the two treatments alone. Among them, 0.1% nZVI + B30 treatment had the best yield increase effect, which was 70.07% higher than that of the control, while 0.1% nZVI + B0 treatment and 0% nZVI + B30 treatment only increased 44.05% and 59.20%, respectively. Accordingly, under Cd3 treatment, the biomass of pakchoi in 0.1% nZVI + B30 treatment increased by 61.66% compared with the control, while the yield of pakchoi in 0.1% nZVI + B0 treatment and 0% nZVI + B30 treatment increased by 40.70% and 59.51% compared with the control, respectively.

#### 2.3.2. Effect of Biochar and Nano Zero-Valent Iron on Physiological Metabolism of Pakchoi

##### Effect of Biochar and Nano Zero-Valent Iron on Chlorophyll Content of Pakchoi

As shown in Table 2, the combined application of BC and nZVI as well as the single application of both significantly increased the chlorophyll content of pakchoi to some extent. Compared with the soil without Cd addition, the total chlorophyll content of pakchoi decreased significantly with the increase in soil Cd content, which decreased by 23.52% and 47.06%, respectively. Under low Cd stress, compared with the control, the total chlorophyll content of pakchoi increased by 42.31% and 23.08%, and the chlorophyll *a* and chlorophyll *b* content increased by 30.23%, 20.93%, 100.00%, and 33.33%, respectively, when BC and nZVI were applied alone. At the same time, the combined application of BC and nZVI significantly enhanced the photosynthetic intensity of pakchoi, that is, with 0.1% nZVI + B30 treatment, the total chlorophyll content of pakchoi increased by 55.77%, chlorophyll *a* and chlorophyll *b* contents increased by 32.56% and 166.67%, respectively. Accordingly, under the condition of Cd3, the total chlorophyll content of pakchoi in 0.1% nZVI + B30 treatment increased by 105.56% compared with the control, and the chlorophyll *a* and chlorophyll *b* contents increased by 75.00% and 350.00%, respectively, the total chlorophyll, chlorophyll *a*, and chlorophyll *b* contents of pakchoi increased by 80.56%, 53.13%, 275.00% and 58.33%, 46.88%, and 150.00%, respectively, in 0% nZVI + B30 treatment and 0.1% nZVI + B0 treatment.

#### 2.3.3. Effect of Biochar and Nano Zero-Valent Iron on Enzyme Activities in Pakchoi Leaves

**Figure 3:**
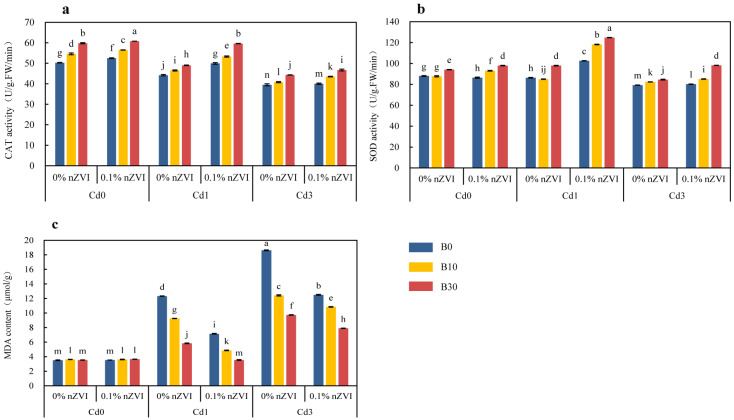
Effect of biochar and nano zero-valent iron addition on catalase (CAT) (**a**), superoxide dismutase (SOD) (**b**), and malondialdehyde (MDA) (**c**) in pakchoi. Data are shown as mean ± SD (n = 3). This means sharing the same letters does not differ significantly at *p* ≤ 0.05. B = Biochar; nZVI = nano zero-valent iron; Cd0 = 0 mg kg^−1^; Cd1 = 1 mg kg^−1^; Cd3 = 3 mg kg^−1^; 0% nZVI = 0% *w*/*w* nZVI; 0.1% nZVI = 0.1% *w*/*w* nZVI; B0 = 0 g kg^−1^; B10 = 10 g kg^−1^; B30 = 30 g kg^−1^.

The addition of Cd to soil significantly increased the MDA content of pakchoi and decreased the activities of CAT and SOD enzymes. Both single and combined applications of BC and nZVI significantly reduced the MDA content of pakchoi and increased the activities of SOD and CAT enzymes to a certain extent (Figure 3a–c). Compared with the treatment without Cd stress, the activities of CAT and SOD enzymes in pakchoi decreased significantly with the increase in soil Cd content, which decreased by 12.13%, 21.47%, 1.92%, and 9.96%, respectively, the MDA content increased by 251.00% and 430.48%. Under low Cd stress, the effects of BC and nZVI independently or in combination on the physiology of pakchoi were different. Specifically, compared with the control when BC and nZVI were applied alone, the activities of CAT and SOD enzymes increased by 10.86%, 13.02%, and 13.44% and 18.73%, respectively, the MDA content decreased by 52.68% and 41.96%. At the same time, the combined application of BC and nZVI significantly enhanced the physiological resistance of pakchoi, and the MDA content of pakchoi significantly decreased by 71.27% when 0.1% nZVI + B30 treatment, the enzyme activities of CAT and SOD increased by 34.86% and 44.57% compared with the control, respectively. Correspondingly, under the condition of Cd3, the MDA content of pakchoi treated with 0.1% nZVI + B30 decreased by 57.57% compared with the control, and the enzyme activities of CAT and SOD increased by 18.23% and 23.86%, respectively, compared with the control. 0% nZVI + B30 treatment and 0.1% nZVI + B0 treatment significantly reduced the MDA content of pakchoi by 47.69% and 32.87%, the enzyme activities of CAT and SOD increased by 12.16%, 1.32%, 6.70%, and 1.19%, respectively, compared with the control.

### 2.4. Synergistic Effect of Biochar and Nano-Sized Zero-Valent Iron on Cd Uptake/Enrichment in Pakchoi

**Figure 4:**
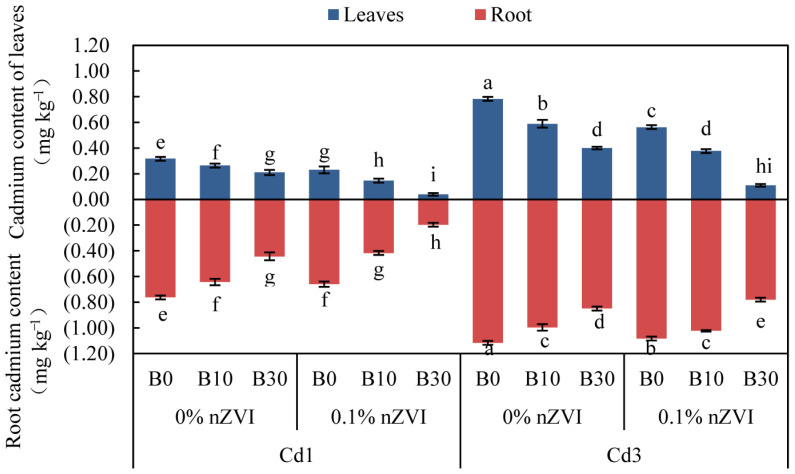
Effect of different treatments on aboveground and underground Cd absorption in pakchoi. Data are shown as mean ± SD (n = 3). Bars sharing the same letters, for a parameter, do not differ significantly at *p* ≤ 0.05. B = Biochar; nZVI = nano zero-valent iron; Cd1 = 1 mg kg^−1^; Cd3 = 3 mg kg^−1^; 0% nZVI = 0% *w*/*w* nZVI; 0.1% nZVI = 0.1% *w*/*w* nZVI; B0 = 0 g kg^−1^; B10 = 10 g kg^−1^; B30 = 30 g kg^−1^.

It can be seen from Figure 4 that the distribution of Cd in pakchoi organs is as follows: root > leaf. The content of Cd in various parts of pakchoi increased significantly with the increase in soil Cd content, but the addition of BC alone and nZVI or the combination of them significantly reduced the content of Cd in the leaves and roots of pakchoi to some extent. Under the condition of low Cd, compared with the control, the Cd content of leaves and roots of pakchoi in 0.1% nZVI + B30 treatment was significantly reduced by 87.50% and 73.68%, respectively, which were lower than the national food safety standard limit (0.2 mg kg^−1^). Other treatments had certain effects on Cd in leaves and roots of pakchoi, which decreased by 53.13%, 28.13%, 34.38%, and 18.75% and 44.74%, 13.16%, 42.11%, and 15.79%, respectively, compared with the control. Correspondingly, with the increase in soil Cd content, that is, under Cd3 stress, 0.1% nZVI + B30 treatment had the best effect on reducing Cd content in leaves and roots of pakchoi, which were significantly lower by 85.90% and 30.36% compared with the control. The improvement effects of other treatments on Cd in leaves and roots of pakchoi were different, which decreased by 51.28%, 28.21%, 48.72%, and 24.36% and 8.93%, 3.57%, 24.11%, and 10.71%, respectively.

### 2.5. Synergistic Effect of Biochar and Nano Zero-Valent Iron on Cd Bioconcentration and Transport Coefficient in Pakchoi

**Figure 5:**
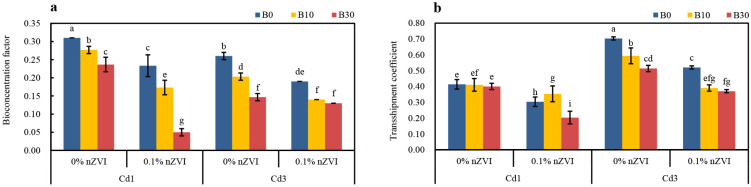
Changes in Cd (**a**) bioconcentration factors and (**b**) transport coefficient under different treatments. Data are shown as mean ± SD (n = 3). Bars sharing the same letters, for a parameter, do not differ significantly at *p* ≤ 0.05. B = Biochar; nZVI = nano zero-valent iron; Cd1 = 1 mg kg^−1^; Cd3 = 3 mg kg^−1^; 0% nZVI = 0% *w*/*w* nZVI; 0.1% nZVI = 0.1% *w*/*w* nZVI; B0 = 0 g kg^−1^; B10 = 10 g kg^−1^; B30 = 30 g kg^−1^.

It can be seen from Figure 5 that the addition of BC and nZVI alone or in combination significantly affected the bioconcentration and transport capacity of Cd in pakchoi to a certain extent (Figure 5a,b). Under the condition of low Cd, the effect of combined treatment of BC and nZVI on reducing Cd bioconcentration and transport capacity of pakchoi is higher than that of BC and nZVI alone. Compared with the control, the bioconcentration coefficient and transport coefficient of 0.1% nZVI + B30 treatment were significantly reduced by 83.87% and 51.22%, respectively. However, the bioconcentration and transport capacity of Cd in pakchoi only decreased by 23.65%, 2.44%, and 24.74% and 26.83% after a single application of BC and nZVI. Correspondingly, with the increase in soil Cd content, that is, under Cd3 stress, the bioconcentration coefficient and transport coefficient of pakchoi decreased significantly by 50.00% and 47.14%, respectively, in 0.1% nZVI + B30 treatment. The bioconcentration and transport capacity of 0% nZVI + B30 treatment and 0.1% nZVI + B0 treatment decreased by 43.58%, 27.14%, and 26.92% and 25.71%, respectively. Generally speaking, compared with the control, the bioconcentration coefficients of Cd in other treatments decreased, and the transport coefficients of Cd in each treatment were also different, and the transport coefficients of each treatment were all less than 1.

### 2.6. Correlation Analysis

**Figure 6:**
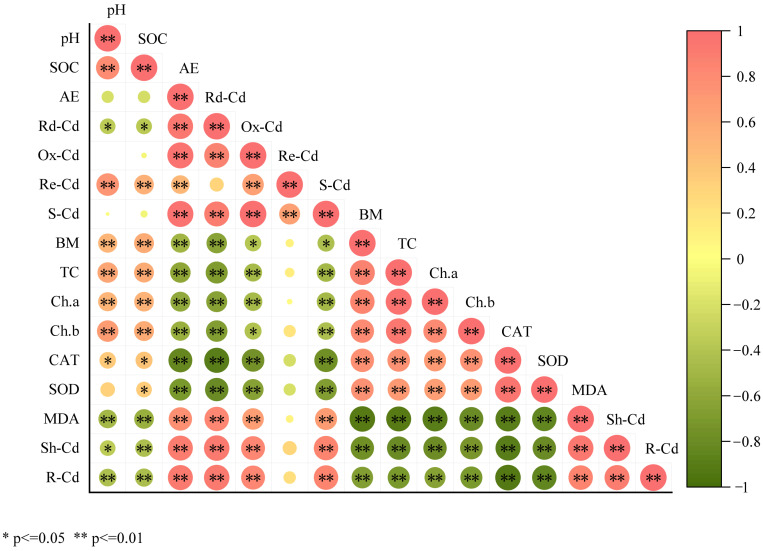
Correlation analysis between soil physicochemical properties and Cd content, physiological indexes, and distribution of Cd in various organs of pakchoi. Data are shown as mean ± SD (n = 3). Note: * and ** in the Table, respectively, indicate *p* ≤ 0.05 (significant), and *p* ≤ 0.01 (highly significant). SOC = soil organic carbon; AE = acid-extractable cadmium; Rd-Cd = Reducible cadmium; Ox-Cd = oxidizable cadmium; Re-Cd = Residual cadmium; S-Cd = total cadmium in soil; BM = total biomass; TC = total chlorophyll; Ch. a = chlorophyll a; Ch. b = chlorophyll b; CAT = catalase; SOD = superoxide dismutase; MDA = malondialdehyde; Sh-Cd = shoot cadmium; R-Cd = cadmium in root.

The relationship between soil physicochemical properties and Cd morphological changes and physiological indexes of pakchoi and Cd morphological distribution in various organs is shown in Figure 6. Among them, soil pH and SOC showed a very significant positive correlation with residual Cd (*p* < 0.01), a significant negative correlation with reducible Cd (*p* < 0.05), and no significant correlation with acid extractable and oxidizable Cd. This shows that increasing soil pH and SOC content can increase the content of residual Cd and decrease the content of reducible Cd. Moreover, soil pH and SOC were significantly negatively correlated with the Cd content in the shoot of pakchoi (*p* < 0.05), and extremely significantly negatively correlated with the Cd content in the root of pakchoi (*p* < 0.01). In addition, the correlation between Cd content in the shoot and root of pakchoi and different forms of Cd in soil reached a very significant level (*p* < 0.01). Specifically, the content of Cd in the shoot and root of pakchoi was positively correlated with acid extractable, reducible, oxidizable, and total Cd. The content of MDA in pakchoi was positively correlated with the content of Cd in shoot and root (*p* ≤ 0.01), (r = 0.935, r = 0.856), there was a very significant negative correlation with biomass, CAT enzyme, SOD enzyme, total chlorophyll, chlorophyll *a*, and chlorophyll *b* content (*p* ≤ 0.01).

### 2.7. Principal Component Analysis (PCA) and Pearson Correlation Matrix

**Figure 7:**
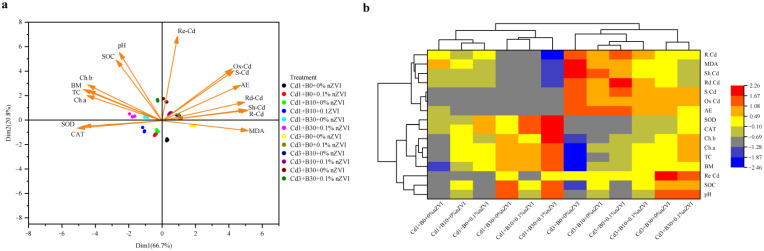
(**a**) Principal component analysis (PCA) and (**b**) heat map of physiological and biochemical parameters under biochar (0, 10, and 30 g kg^−1^) and nano zero-valent iron (0% and 0.1%, *w*/*w*) applications.

Principal component analysis (PCA) and Pearson correlation matrix indicated that the variables were significantly correlated between different treatments (Figure 7a,b). The two dimensions of PCA, namely Dim-1 and Dim-2, synergistically explain nearly 86% of the variability in the dataset. Dim-1 contributed 66.7%, while Dim-2 accounted for 20.8% of the total (Figure 7a). There is a significant positive correlation between soil-reducible Cd, Cd content, and MDA concentration level in shoot parts of pakchoi (Figure 7b). The biomass and total chlorophyll content of pakchoi showed a positive correlation in the combined treatment of biochar and nano zero-valent iron (Figure 7b). In addition, there was a significant negative correlation between Cd content in pakchoi roots, total chlorophyll content, and biomass (Figure 7b). Principal component analysis of pakchoi under Cd stress and different levels of amendments PCA-biplot expressing the interactions between different attributes of the treatments (Figure S1).

PCA-loading plots were used to evaluate chlorophyll content, antioxidant enzyme activity, and Cd uptake content in pakchoi tissues treated with biochar and nano-zero-valent iron in Cd-contaminated soil. Variables included R.Cd (root cadmium), MDA (malondialdehyde), Sh Cd (shoot cadmium), Rd Cd (reducible cadmium), S Cd (soil total cadmium), Ox Cd (oxidizable cadmium), AE (acid extractable cadmium), SOD (superoxide dismutase), CAT (catalase), Ch.b (chlorophyll b), Ch.a (chlorophyll a), TC (total chlorophyll), BM (total biomass), Re Cd (residual cadmium), SOC (soil organic carbon), and pH. B = Biochar; nZVI = nano zero-valent iron; Cd0 = 0 mg kg^−1^; Cd1 = 1 mg kg^−1^; Cd3 = 3 mg kg^−1^; 0% nZVI = 0% *w*/*w* nZVI; 0.1% nZVI = 0.1% *w*/*w* nZVI; B0 = 0 g kg^−1^; B10 = 10 g kg^−1^; B30 = 30 g kg^−1^.

## 3. Discussion

This study confirmed that the combined application of biochar (BC) and nano zero-valent iron (nZVI) could help to restore cadmium (Cd) contaminated soil, reduce Cd bioavailability and improve the growth status of pakchoi. The background of this research stems from the fact that industrial and agricultural activities (mineral mining, industrial waste discharge, and intensive aquaculture manure application) caused by early urban development aggravated farmland Cd pollution [42], superimposed on the lag of environmental supervision in the process of urbanization and the excessive application of agricultural chemicals (such as Cd-containing phosphate fertilizer), resulted in a significant increase in soil Cd ecological risk [43,44]. When the content of soil Cd exceeds the safe threshold, the enhancement of its bioavailability inhibits the photosynthetic efficiency and antioxidant system of plants and enters the human body through the food chain, which poses a threat to human safety [45,46]. Previous studies have reported several times that the separate application of BC and nZVI can promote plant growth and remediation of heavy metal-contaminated soil [35,47]. However, the synergistic effect of the combined application of the two on the regulatory pathway of soil heavy metal Cd transport and plant development has not been clearly defined. In this study, biochar (BC) and nano-zero-valent iron (nZVI) were used as materials to explore the regulatory effects of independent and combined use of BC and nZVI on heavy metal Cd in plants and the mechanism of heavy metal Cd migration and transformation process in soil–plant system through pakchoi pot experiment.

This study found that higher concentrations of Cd (3 mg kg^−1^) severely hindered the growth and physiological metabolism of pakchoi (Figure 2 and Figure 3, Table 2). This is consistent with what Abbas et al. observed in wheat [48]. In fact, as a non-essential element of plants, Cd reduces the ability of plant roots to penetrate the soil and to obtain soil surface water and nutrients [49], and Cd toxicity affects root growth by inhibiting root cell division and elongation or prolonging the cell cycle, thus limiting the development of new shoots and leading to the decrease in plant biomass [50]. Adding a certain amount of BC (30 mg kg^−1^) to Cd-contaminated soil greatly inhibited the mobility and toxicity of Cd and improved the overall morphological characteristics of pakchoi (Figure 2). It is reported that the porous structure on its surface immobilizes Cd through physical adsorption, while oxygen functional groups (OFGs) form stable coordination compounds with Cd, synergistically reducing the bioavailability of Cd, promoting plant root vitality, and ultimately improving crop productivity [51,52]. Photosynthetic pigments (total chlorophyll, chlorophyll *a*, and chlorophyll *b*) are responsible for absorbing light energy at specific wavelengths required by plants for photosynthesis [53], and their activity is somewhat reduced in pakchoi (Table 2). This results from Cd-induced damage to chloroplast ultrastructure (disintegration of basal lamellar) and inhibition of chlorophyll synthesis, resulting in a significant decrease in photosynthetic electron transfer efficiency and carbon assimilation ability, reducing the synthesis of photosynthetic products, resulting in yellowing of plant leaves [54]. In this study, compared with the control, each treatment increased the photosynthetic pigment content of pakchoi leaves, and the combined effect of combined application of BC and nZVI was more obvious (Table 2). This indicates that the addition of BC and nZVI to Cd-contaminated soil can improve the ultrastructure of plants, which is beneficial to the growth and development of plants. The application of BC to many plant species such as maize and rice under Cd stress increased their photosynthetic efficiency [55,56]. As the final product of lipid membrane peroxidation, MDA is an important physiological index to measure the response of plants to heavy metal stress [57]. High concentration of Cd in pearl millet increased the content of reactive oxygen species (ROS) and malondialdehyde (MDA) in different parts by reducing photosynthesis, causing severe oxidative damage [58]. In this study, it was found that the combined application of BC and nZVI reduced the MDA content of pakchoi leaves under heavy metal stress, indicating that the addition of BC and nZVI alleviated the oxidative damage of pakchoi leaves. In this study, it was found that a higher concentration of Cd (3 mg kg^−1^) increased the MDA content of pakchoi leaves and exacerbated the oxidative damage suffered by plants. Studies have shown that when plants are stressed by heavy metals, reactive oxygen species (ROS) produced in the body are the main substances that lead to the oxidation of biological macromolecules such as proteins and nucleic acids, thus damaging cell integrity [59]. In order to maintain reactive oxygen species homeostasis, plants respond to the negative effects caused by heavy metal stress through antioxidant enzyme systems such as superoxide dismutase (SOD) and catalase (CAT) [60]. In this study, both SOD and CAT activities in pakchoi leaves showed a decreasing trend with the increase in Cd concentration compared with the control group. However, both SOD and CAT activities in pakchoi leaves showed an upward trend after the application of BC and nZVI. nZVI was also able to capture OH and form a FeO (OH) wrapping layer on the surface of plant roots, thus reducing the expression of membrane lipid peroxidation, decreasing the MDA content in pakchoi leaves, and providing Fe^2+^/Fe^3+^ as the prosthetic group of enzymes such as CAT and SOD, promoting the synthesis of antioxidant enzymes [61]. When BC was combined with nZVI, BC decreased the effectiveness of Cd and decreased the generation of ROS, while nZVI supplemented the necessary Fe and improved the structural stability and activity of soil enzymes, thus maintaining the normal levels of SOD and CAT more effectively [62]. Therefore, the combination of BC and nZVI inhibited the oxidative damage of pakchoi leaves caused by soil Cd pollution.

According to bioavailability, the occurrence forms of Cd in soil can be sorted as acid extractable state > reducible state > oxidizable state > residual state [63]. This study found that the distribution of various forms of Cd was different in different treatments. Adding BC alone or combined with nZVI significantly increased the content of residual Cd and decreased the content of acid-extractable Cd (Figure 1). However, there was no significant difference in Cd morphological changes between BC alone and the combined administration of BC and nZVI, which indicated that the above change trend was mainly related to BC. BC significantly increased the pH and SOC content of Cd-contaminated soil (Table 1), and pH and SOC were important factors affecting the occurrence forms of heavy metals in soil [64]. The increase in soil pH is related to the alkaline nature of BC (Table S1), and its alkaline cations are converted into oxides, hydroxides, and carbonates during pyrolysis. The dissolution of these alkaline substances in biochar increases soil pH, thus accelerating the hydrolysis of metal cations, promoting the precipitation formation of metal (oxygen) hydroxides, at the same time, it can also enhance the ion exchange effect on the BC surface and reduce the mobility of soil Cd [65]. In addition, BC is rich in stable carbon, and the addition of BC can also be used as a stable carbon source for soil while increasing soil SOC content [66]. Some studies have shown that group forms such as humus, −SH, and −NH_2_ in soil can form stable complexes with heavy metals in soil, thus reducing the migration ability of heavy metals in soil [67]. It was also observed in this study that the addition of BC significantly improved the nutrient content of the soil (Table S5). As found by Zhao et al., the application of BC in Cd-contaminated soil improved the C, N, K, Ca, and P contents in alfalfa soil and plant biomass [68]. Therefore, the soluble nutrients present in BC can be used as a source of soil nutrients and improve the productivity of many crops.

In this study, the Cd content of various organs of pakchoi was different, and the specific manifestation was root > shoot (Figure 4). When Cd^2+^ reaches the epidermis of plant roots, it is trapped by the cell wall through the apoplast pathway, which alleviates the biotoxicity of Cd in the initial stage [69]. Cd^2+^ was subsequently compartmentalized in vacuoles by aerial mesophyll cells and combined with phytochelating peptides (PCs) to achieve re-detoxification [70]. The application of BC and nZVI had significant effects on the distribution of Cd, the enrichment and transport of Cd in pakchoi, and the cumulative effect of the combined application of the two was more significant than that of the single application (Figure 4 and Figure 5). Because of the smaller particle size and the increase in specific surface area, BC provides additional adsorption sites to adsorb exchanged Cd, thus limiting the bioavailability of Cd, and its alkaline component promotes Cd to generate carbonate and hydroxide precipitation, which makes the proportion of residual Cd rise [71]. The PAH layer of BC can also provide electrons for the reduction in Cd [72]. Similarly, nZVI enhances the electrostatic adsorption of Cd^2+^ through surface charge action, thereby converting Cd from a usable form to an immobilized form [73]. Through surface reduction, part of Cd^2+^ was reduced to Cd0, and FeOOH colloid achieved co-precipitation, and the formation of Cd-organic complex was inhibited by competition, reducing the bioavailability of Cd [36]. When they were applied together, BC was used as the carrier of nZVI, which improved the dispersion of nZVI decreased its oxidation degree, and made it contact with Cd in soil more effectively [74]. The synergistic effect of BC and nZVI provides improved surface area, adsorption characteristics, and mobility in soil [75,76].

All in all, the addition of BC maximizes the improvement of soil physicochemical and biological properties and reduces the availability of Cd and its accumulation in plants and toxicity [77]. The strong reducibility of nZVI is one of the main mechanisms of its efficiency [78]. BC and nZVI showed synergistic effects on the improvement of Cd-contaminated soil and the inhibition of Cd uptake by pakchoi, which may be related to factors such as crop variety, nanomaterials and biochar application rates, and various Cd resistance mechanisms. However, it should be noted that the long-term remediation efficiency of BC was attenuated in field experiments, and the aging process of nZVI caused the reactivation of Cd [79]. Therefore, in the subsequent soil remediation work, more research is needed on BC and nZVI in different climate zones and soil environments, as well as application technology and material innovation, to further clarify the application value of the two in the field of soil remediation.

## 4. Materials and Methods

### 4.1. Soil Samples and Soil Amendments

Soil samples were collected from 0 to 20 cm surface layer of farmland in Lanzhou City, Gansu Province (36.061° N, 103.834° E, 1518 m above sea level), sandy loam texture, carbonate-free alkaline soil. After manually removing stones and plant residues, they are naturally air-dried through a 2 mm sieve for later use. The soil basic fertility indexes were total nitrogen 0.55 g kg^−1^, available phosphorus 25.16 mg kg^−1^ and available potassium 100.14 mg kg^−1^ (see Table S1 for other physical and chemical parameters). For cadmium pollution treatment, cadmium sulfate octahydrate (CdSO_4_·8H_2_O, analytical grade) solution is added at one time. The dosage is calculated based on the total amount of soil in the pot test and stirred while adding to ensure uniform mixing. The contaminated soil is sealed with tin foil paper and punctured to ensure ventilation and control water evaporation and aged in the dark for 4 weeks under the condition of keeping the field water holding capacity of 60–70% (during this period, the water content is maintained by weighing method). Finally, the aged soil is evenly mixed and air-dried for later use.

Biochar (BC) was provided by Liaoning Jinhefu Agricultural Science and Technology Co., Ltd. (Liaoning, China), and its preparation method was as follows: corn (*Zea mays* L.) straw was placed under oxygen-limiting conditions and then pyrolyzed at 500 °C for 2 h. The main physical and chemical properties were shown in Table S2. The microscopic morphology of the sieved biochar samples (particle size ≤ 2 mm) was analyzed by scanning electron microscope, and the crystal structure characteristics were determined by X-ray diffractometer. The characterization results are detailed in Figure S1. Please refer to Chinese-authorized patent CN102092709B for specific preparation process parameters [80]. Nano zero-valent iron (nZVI) was purchased from Institute of Nanomaterials, Gansu Academy of Sciences. The material has a specific surface area of 17.58 m^2^g^−1^ and a true density of 0.6 g cm^3^ (based on supplier technical parameters). X-ray diffraction (XRD) was used to analyze its phase composition, and the characterization results are detailed in Figure S2. The liquid suspension was prepared as follows: (1) nZVI suspension: nano zero-valent iron (nZVI) particles were mixed with ultrapure water and sonicated for 30 min using a Branson 5800 sonicator (Dongguan Branson Ultrasonic Equipment Co., Ltd., Guangdong, China) to promote dispersion; (2) BC suspension: biochar (BC) powder was mixed with ultrapure water and ultrasonicated to form a homogeneous system; (3) BC-nZVI treatment solution: Biochar (BC) powder was added to the nZVI suspension and continued stirring until thoroughly mixed. All suspensions are prepared immediately prior to use, ensuring the freshness of the treatment.

The test plant Pakchoi (*B. rapa* subsp. *chinensis* ‘Shanghaiqing’) was purchased from Weifang, China, Demin Seed Industry Co., Ltd.

### 4.2. Plant Growth Experiment

The pot experiment was carried out in the greenhouse of the College of Resources and Environment of Gansu Agricultural University from May to July 2024 (with a canopy to prevent rain erosion). The test set up a three-factor gradient: biochar (BC) 0, 10, 30 g kg^−1^ (labeled B0, B10, B30); nano zero-valent iron (nZVI) 0%, 0.1% (*w*/*w*, on dry soil basis, labeled as 0% nZVI, 0.1% nZVI); cadmium (Cd) 0, 1, 3 mg kg^−1^ (labeled Cd0, Cd1, Cd3). A 3 × 2 × 3 completely combined design was used with 18 treatment groups in 3 replicates (54 pots in total), completely randomized. Use a polyethylene pot (diameter 23 cm × height 12 cm) to hold 2.5 kg (dry weight) of Cd contaminated soil, add BC and nZVI in turn according to each treatment gradient, and then mix base fertilizer (add N 0.2 g, P_2_O_5_ 0.15 g and K_2_O 0.2 g per kg soil, derived from urea ≥ 46%, potassium dihydrogen phosphate ≥ 52%, potassium sulfate ≥ 54%, *w*/*w*, respectively). After balancing for 3 days, sow, leave 3 seedlings in each pot, and maintain the soil water content at 60–70% of the field water holding capacity by weighing method every day. Plant and soil samples were collected after 40 days of culture (see Table S3 for details).

### 4.3. Measurement Items and Methods

#### 4.3.1. Determination of the Physiological Index of Pakchoi

After harvesting, pakchoi was washed with ultrapure water, dried with absorbent paper, and divided into leaf and root parts to record the fresh weight, while liquid nitrogen quick-freezing was used for the determination of physiological indexes. The activities of malondialdehyde (MDA) and catalase (CAT) were determined by the thiobarbituric acid method and ultraviolet absorption method, respectively [81,82]. The activity of superoxide dismutase (SOD) and total chlorophyll were determined by nitrogen blue tetrazole photoreduction and acetone extraction spectrophotometry, respectively [83,84].

#### 4.3.2. Determination of Cd Content in Pakchoi

After the pakchoi plants were harvested, they were washed twice with tap water and high-purity water, respectively, the water was dried and the plants were divided into above-ground and underground parts, which were baked in a drying oven at 105 °C for 30 min, then baked at 65 °C to constant weight, and the dry biomass was recorded, then the sample was ground to powder, and 0.2000 g of the sample was accurately weighed and digested in the mixture of HNO_3_:H_2_O_2_ (7:1), and the Cd content was determined by inductively coupled plasma emission spectrometer (ICP-5000, Beijing, China, Concentrator Technology Co., Ltd.) [85], LOD (Limit of detection) = 1 µg/L.

#### 4.3.3. Determination of Soil Physical and Chemical Properties

Soil samples were collected from rhizosphere soil, air-dried at room temperature (25 °C), ground, sieved, sealed, and stored for further testing. Soil pH: the water–soil ratio is 2.5:1, mix and shake well, and measure with an acidity meter (PB-10, Guangzhou, China, Boletai Technology Co., Ltd.); soil organic carbon: accurately weigh 0.3500 g of dry soil and put it into a 150 mL triangular flask, add 5 mL of potassium dichromate solution, then add 5 mL of concentrated sulfuric acid, cover a curved funnel and place it on an electric heating plate at 180 °C for 30 min, take it out and cool, then add 2 to 3 drops of ortho-phanoline indicator, titrate with 0.2 mol L^−1^ FeSO_4_, and record the titration number; total nitrogen was digested by Kjeldahl method in soil samples and determined by Kjeldahl analyzer; available phosphorus: accurately weigh 2.5000 g of dried soil and put it into a 100 mL triangular flask, add 0.50 g of activated carbon, then add 50 mL of 0.5 mol L^−1^ sodium bicarbonate solution, shake for 30 min, take the filtrate to the volumetric flask, then add 5 mL of molybdenum antimony developer, constant volume to 50 mL, and accurately measure at a wavelength of 880 nm using a spectrophotometer; available potassium: put 5.00 g of dry soil into a 150 mL triangular flask, then add 50 mL of 1 mol L^−1^ neutral ammonium acetate (NH_4_OAc) solution, shake the mixture for 30 min and filter it through qualitative filter paper, and measure it on a flame photometer together with the potassium standard solution [86].

#### 4.3.4. Determination of Different Fractions and Total Cd in Rhizosphere Soil

The total Cd content in rhizosphere soil was determined by microwave digestion. The soil sample is air-dried soil. After grinding through a 100-mesh sieve, accurately weigh 0.1000 g of the soil sample and put it into a PVC digestion tube. After adding mixed acid (HNO_3_:HCl:HF = 6:2:1), it is moved into the stage and digestion begins. After the digestion is completed, lower the temperature, move the digestion tank to the fume hood in turn, heat (130–150 °C) to drive the acid until the color disappears, cool, and transfer to a 50 mL volumetric flask. The extracts were determined by an inductively coupled plasma emission spectrometer (ICP-5000, Beijing Concentrator Technology Co., Ltd.), LOD (Limit of detection) = 1 µg/L. Different forms of Cd were extracted by the BCR continuous extraction method, and then the content of Cd was determined by inductively coupled plasma emission spectrometer (ICP-5000, Beijing Concentrator Technology Co., Ltd.) [87], LOD(Limit of detection) = 1 µg/L. See Table S4 for specific steps.

#### 4.3.5. Calculation of Bioaccumulation and Transport Coefficient

The bioconcentration factor (BCF) was calculated as the ratio of the Cd content in plant tissue to the soil Cd content [88]. The metal transport coefficient (TF) was calculated as the ratio of the Cd content in the above-ground plant part of the plant to the Cd content of the underground plant part.

### 4.4. Data Analysis and Processing

In this study, Microsoft Excel 2016 (data collation) and IBM SPSS 27.0.1 (statistical modeling) was used to analyze the observed data. To comprehensively evaluate the effects of Cd on the physiological and biochemical responses of pakchoi, an analysis of variance (one-way ANOVA) was performed between different treatments. Tukey’s test was used for multiple comparisons to assess significance (*p* < 0.05), and the results were shown as the mean ± mean square error (SD) of three replicates, where different letters indicate a significant difference of *p* < 0.05. Correlation analysis was performed using OriginPro 2021 to determine the linear relationship between soil physicochemical properties, Cd content, and physiology and biochemistry of pakchoi plants. Principal component analysis (PCA) was used to analyze the relationship between growth parameters and physiological traits, and the significance thresholds were set as *p* < 0.05 and *p* < 0.01.

## 5. Conclusions

While numerous studies are being conducted in the search for new technologies and materials to remediate agricultural soils contaminated by heavy metals, the problem of cadmium (Cd) contamination still poses a serious threat to human health. Under the conditions of this experiment, the combined application of biochar (BC) and nano-zero-valent iron (nZVI) helps restore the growth of pakchoi in cadmium (Cd)-contaminated soil and significantly reduces the biological toxicity of Cd to a certain extent. Therefore, the combined application of the two can be considered as soil amendments to achieve in situ stabilization of contaminated sites. At the same time, in future soil remediation practices, it is recommended to implement long-term localization experiments based on multi-environmental factors similar to those in this study in different soil types and different crop varieties as well as in the long-term stability of amendments to improve the decontamination efficiency of BC and screen out reasonable nZVI concentrations to achieve specific soil remediation goals. In short, it is still necessary to develop a systematic field remediation technology system to deal with the adverse effects of Cd-contaminated soil on sustainable agricultural development.

## Figures and Tables

**Table 1:** Effect of BC and nZVI addition on soil pH and SOC content.

Treatments		Cd0		Cd1		Cd3	
		pH	SOC (g kg^−1^)	pH	SOC (g kg^−1^)	pH	SOC (g kg^−1^)
0% nZVI	B0	8.20 ± 0.02 gh	5.83 ± 0.68 f	8.23 ± 0.03 fg	5.88 ± 0.33 f	8.24 ± 0.01 fg	5.79 ± 0.24 f
	B10	8.25 ± 0.03 efg	7.09 ± 0.33 cd	8.32 ± 0.03 cd	7.00 ± 0.39 de	8.33 ± 0.05 c	7.07 ± 0.41 cd
	B30	8.62 ± 0.03 b	8.23 ± 0.74 a	8.63 ± 0.06 b	8.05 ± 0.86 ab	8.66 ± 0.02 ab	7.98 ± 0.30 ab
0.1% nZVI	B0	8.18 ± 0.06 h	5.93 ± 0.50 f	8.21 ± 0.04 gh	5.90 ± 0.71 f	8.27 ± 0.01 ef	6.18 ± 0.45 ef
	B10	8.27 ± 0.03 def	6.89 ± 0.34 de	8.29 ± 0.04 cde	6.95 ± 0.34 de	8.30 ± 0.03 cde	7.26 ± 0.76 bcd
	B30	8.61 ± 0.03 b	7.90 ± 0.66 abc	8.62 ± 0.06 b	8.19 ± 0.51 a	8.69 ± 0.03 a	8.01 ± 0.72 ab

Data are shown as mean ± SD (n = 3). This means sharing the same letters, for a parameter, does not differ significantly at *p* ≤ 0.05. SOC = Soil organic carbon; B = Biochar; nZVI = nano zero-valent iron; Cd0 = 0 mg kg^−1^ (Cd concentration is <LOD); Cd1 = 1 mg kg^−1^; Cd3 = 3 mg kg^−1^; 0% nZVI = 0% *w*/*w* nZVI; 0.1% nZVI = 0.1% *w*/*w* nZVI; B0 = 0 g kg^−1^; B10 = 10 g kg^−1^; B30 = 30 g kg^−1^.

**Table 2:** Effect of biochar and nano zero-valent iron on chlorophyll content of pakchoi.

Treatments		Cd0			Cd1			Cd3		
		Total Chlorophyll (mg g^−1^ FW)	Chlorophyll *a* (mg g^−1^ FW)	Chlorophyll *b* (mg g^−1^ FW)	Total Chlorophyll (mg g^−1^ FW)	Chlorophyll *a* (mg g^−1^ FW)	Chlorophyll *b* (mg g^−1^ FW)	Total Chlorophyll (mg g^−1^ FW)	Chlorophyll *a* (mg g^−1^ FW)	Chlorophyll *b* (mg g^−1^ FW)
0% nZVI	B0	0.68 ± 0.01 defg	0.54 ± 0.02 cdef	0.14 ± 0.02 cdef	0.52 ± 0.01 i	0.43 ± 0.03 h	0.09 ± 0.02 g	0.36 ± 0.04 j	0.32 ± 0.03 i	0.04 ± 0.01 h
	B10	0.72 ± 0.04 cde	0.56 ± 0.03 bcd	0.16 ± 0.04 bcde	0.63 ± 0.03 gh	0.50 ± 0.02 efg	0.13 ± 0.02 defg	0.54 ± 0.02 i	0.45 ± 0.02 gh	0.09 ± 0.03 g
	B30	0.77 ± 0.02 abc	0.60 ± 0.03 ab	0.17 ± 0.04 bcd	0.74 ± 0.09 bcd	0.56 ± 0.04 bcd	0.18 ± 0.07 bc	0.65 ± 0.05 efh	0.49 ± 0.05 fg	0.15 ± 0.01 bcde
0.1% nZVI	B0	0.74 ± 0.04 bcd	0.57 ± 0.04 bcd	0.17 ± 0.01 bcde	0.64 ± 0.05 fgh	0.52 ± 0.04 def	0.12 ± 0.02 efg	0.57 ± 0.07 hi	0.47 ± 0.04 gh	0.10 ± 0.03 fg
	B10	0.75 ± 0.10 bc	0.57 ± 0.05 bcd	0.18 ± 0.05 bc	0.71 ± 0.04 cdef	0.55 ± 0.01 bcde	0.16 ± 0.03 bcde	0.62 ± 0.03 gh	0.50 ± 0.03 fg	0.13 ± 0.02 defg
	B30	0.83 ± 0.04 a	0.64 ± 0.03 a	0.20 ± 0.02 ab	0.81 ± 0.09 ab	0.57 ± 0.05 bc	0.24 ± 0.04 a	0.74 ± 0.01 bcd	0.56 ± 0.03 bcd	0.18 ± 0.02 bc

This means sharing the same (normal) letters (a to j), for a parameter, does not differ significantly at *p* ≤ 0.05. B = Biochar; nZVI = nano zero-valent iron; Cd0 = 0 mg kg^−1^; Cd1 = 1 mg kg^−1^; Cd3 = 3 mg kg^−1^; 0% nZVI = 0% *w*/*w* nZVI; 0.1% nZVI = 0.1% *w*/*w* nZVI; B0 = 0 g kg^−1^; B10 = 10 g kg^−1^; B30 = 30 g kg^−1^. Data are shown as mean ± SD (n = 3).

## Data Availability

The original contributions presented in this study are included in the article/Supplementary Materials, further inquiries can be directed to the corresponding author.

## Supplementary Materials

The following supporting information can be downloaded at https://www.mdpi.com/article/10.3390/ijms26094373/s1.

## Abbreviations

The following abbreviations are used in this manuscript:

AE	Acid extractable
BCF	Bioconcentration factor
BCR	Community Bureau of Reference
BM	Total biomass
CAT	Catalase
Cd	Cadmium
Ch. a	Chlorophyll a
Ch. b	Chlorophyll b
H_2_O_2_	Hydrogen-peroxide;
ICP-5000	Inductively coupled plasma emission spectrometer, Beijing condenser technology Co., Ltd. Beijing, China
MDA	Malondialdehyde
Ox-Cd	Oxidizable cadmium
Re–Cd	Residual cadmium
R–Cd	Cadmium in root
Rd-Cd	Reducible cadmium
ROS	Reactive oxygen species
SOD	Superoxide dismutase
TBA	Thiobarbituric acid
TCA	Trichloroacetic acid
TC	Total chlorophyll
TF	Metal transport coefficient
Sh-Cd	Shoot cadmium
SOC	Soil organic carbon
S–Cd	Cadmium in soil
