# Supplementary material for: Study on the Interaction Effect of Heavy Metal Cadmium in Soil–Plant System Controlled by Biochar and Nano-Zero-Valent Iron
Source: Int J Mol Sci. 2025 May 4;26(9):4373. doi: 10.3390/ijms26094373 (PMC12072827; doi:10.3390/ijms26094373)
Supplement: Supplementary file 1 [file ijms-26-04373-s001.zip › ijms-3604406-supplementary.pdf]

International Journal of
Molecular Sciences

Table S1. Basic physical and chemical properties of the experimental soil.

Indices	pH	SOC ($g\ kg^{-1}$)	Total N ($g\ kg^{-1}$)	Available P ($mg\ kg^{-1}$)	Available K ($mg\ kg^{-1}$)	Total Cd ($mg\ kg^{-1}$)
Value	8.20	7.88	0.85	27.16	107.14	<LOD

SOC= Soil organic carbon; N= Nitrogen; P= Phosphorous; K= Potassium; Cd= Cadmium; LOD=Limit of detection.

Table S2. Basic properties of biochar.

Cracking temperature (℃)	pH	Element content (%)						Ash content (%)	Specific surface area (m^2g^{-1})	Cd content ($mg\ kg^{-1}$)
		C	N	P	K	Ca	Mg			
500	9.21	53.28	1.04	0.26	0.51	0.80	0.47	35.64	11.30	<LOD

C= Carbon; N= Nitrogen; P= Phosphorous; K= Potassium; Ca= Calcium; Mg= Magnesium; Cd= Cadmium; LOD=Limit of detection.

Table S3. Experimental design.

Treatment	Treatment group	Cadmium ($mg\ kg^{-1}$)	Biochar ($g\ kg^{-1}$)	Nano zero-valent iron (%)
1	Cd0+B0+0%nZVI	0	0	0
2	Cd0+B10+0%nZVI	0	10	0
3	Cd0+B30+0%nZVI	0	30	0
4	Cd0+B0+0.1%nZVI	0	0	0.1
5	Cd0+B10+0.1%nZVI	0	10	0.1
6	Cd0+B30+0.1%nZVI	0	30	0.1
7	Cd1+B0+0%nZVI	1	0	0
8	Cd1+B10+0%nZVI	1	10	0
9	Cd1+B30+0%nZVI	1	30	0
10	Cd1+B0+0.1%nZVI	1	0	0.1
11	Cd1+B10+0.1%nZVI	1	10	0.1
12	Cd1+B30+0.1%nZVI	1	30	0.1
13	Cd3+B0+0%nZVI	3	0	0
14	Cd3+B10+0%nZVI	3	10	0
15	Cd3+B30+0%nZVI	3	30	0
16	Cd3+B0+0.1%nZVI	3	0	0.1
17	Cd3+B10+0.1%nZVI	3	10	0.1
18	Cd3+B30+0.1%nZVI	3	30	0.1

B = Biochar; nZVI = nano zero-valent iron; Cd0 = 0 mg kg^{-1}; Cd1 = 1 mg kg^{-1}; Cd3 = 3 mg kg^{-1}; 0% nZVI = 0% w/w nZVI; 0.1% nZVI = 0.1% w/w nZVI; B0 = 0 g kg^{-1}; B10 = 10 g kg^{-1}; B30 = 30 g kg^{-1}.

https://doi.org/10.3390/ijms26094373

Table S4. Determination of various forms of cadmium in soil by Community Bureau of Reference method.

procedure	Cd morphology	Extraction procedure
1	Acid extractable state	0.11 mol L^{-1} HOAc 40 mL; acetic acid) per 1 g of dry soil sample was shaken for 16 h on a mechanical shaker at 25°C.
2	Reducible state	0.5 mol L^{-1} NH_2OH-HCl (40 mL; adjusted to pH 1.5 with HNO_3) was added to residue and shaken for 16 h at 25°C.
3	Oxidizable state	8.8 mol L^{-1} H_2O_2 (10 mL) was added to the residue and digested for 1 h at 25°C, and then for 1 h at 85°C in a water bath with a second volume of H_2O_2. The solution was then evaporated to a few mL. 1 mol L^{-1} NH_4OAc (50 mL; adjusted to pH 2.0 with HNO_3) was added to residue and shaken for 16 h at 25°C.
4	Residual state	HNO_3-HF-$HClO_4$ digestion, the same procedure as of the total metal determination was followed.

Table S5. Effects of biochar and nano zero-valent iron on soil total nitrogen, available phosphorus, and available potassium.

Treatments		Cd0			Cd1			Cd3		
		Total N (g kg^{-1})	Available P (mg kg^{-1})	Available K (mg kg^{-1})	Total N (g kg^{-1})	Available P (mg kg^{-1})	Available K (mg kg^{-1})	Total N (g kg^{-1})	Available P (mg kg^{-1})	Available K (mg kg^{-1})
0%	B0	0.61±0.11efg	28.78±2.67g	107.70±0.90i	0.62±0.05ef	32.81±0.29efg	107.74±0.10i	0.51±0.05g	32.30±4.15efg	107.11±0.43i
nZVI	B10	0.67±0.12def	44.28±2.48c	114.90±1.98fg	0.68±0.03cdef	42.53±4.26cd	113.13±1.35g	0.60±0.05fg	43.20±2.15cd	111.74±2.72gh
	B30	0.74±0.06abcd	51.81±11.70b	131.54±2.09ab	0.81±0.01ab	51.90±3.39b	127.66±3.13bc	0.75±0.06abcd	60.81±4.04a	122.13±0.31de
0.1%	B0	0.62±0.05ef	32.44±0.71efg	108.43±0.99hi	0.60±0.06fg	31.02±4.98fg	108.85±0.51hi	0.51±0.01g	36.79±2.64def	108.76±0.70hi
nZVI	B10	0.69±0.02cdef	40.28±2.69cd	118.56±0.14ef	0.71±0.04bcde	43.45±3.66cd	114.77±3.76fg	0.62±0.03ef	39.01±4.97cde	114.75±4.45fg
	B30	0.78±0.04abc	61.96±6.64a	134.83±7.27a	0.84±0.15a	63.77±7.25a	129.55±1.35bc	0.77±0.07abcd	59.70±1.75a	125.93±3.21cd

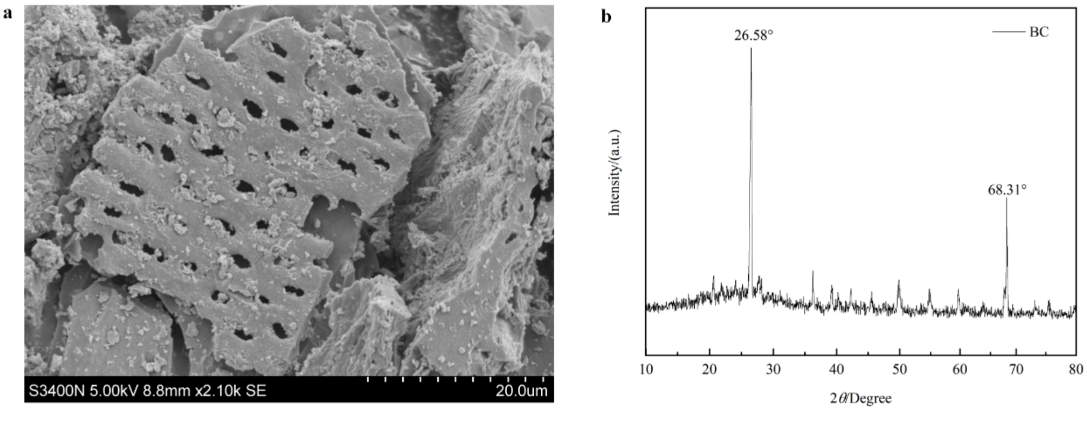

Figure S1. SEM image (**a**) and XRD image (**b**) of the biochar used in this experiment.

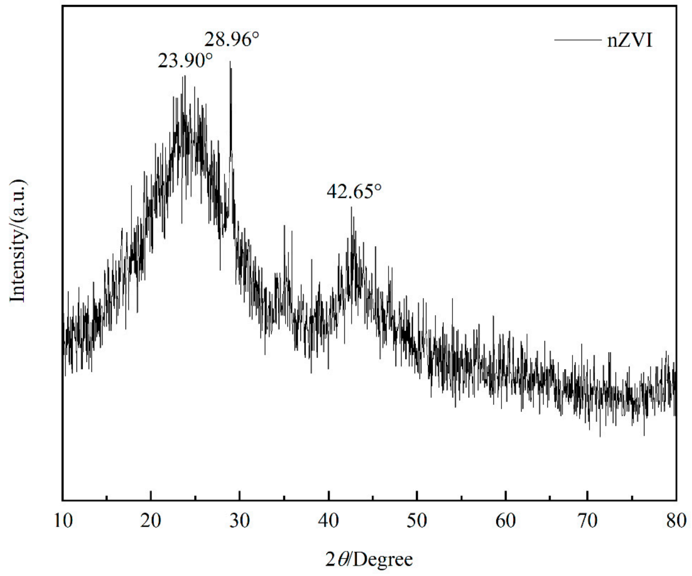

Figure S2. XRD images of nano zero-valent iron used in this experiment.

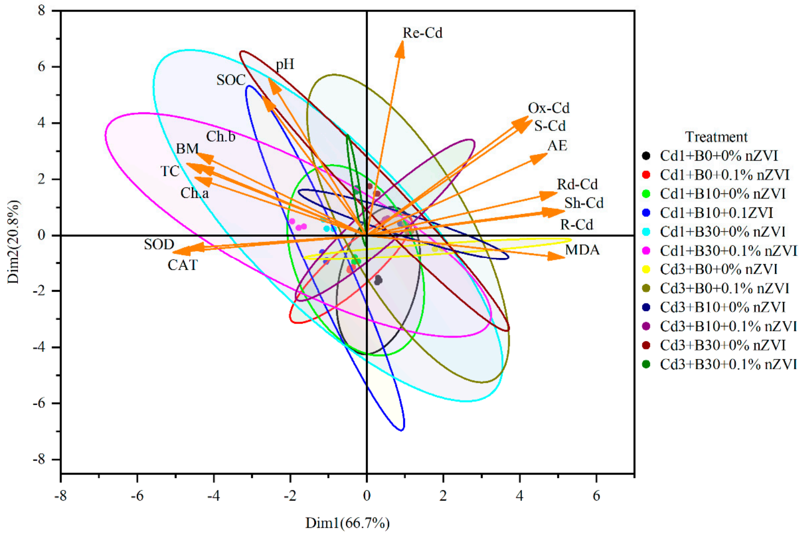

Figure S3. Principal component analysis of plant (*Brassica rapa* L. subsp. *chinensis*) under Cd stress and different levels of amendments PCA-biplot expressing the interactions between different attributes of the with treatments. B = Biochar; nZVI = nano zero-valent iron; Cd0 = 0 mg kg^{-1}; Cd1 = 1 mg kg^{-1}; Cd3 = 3 mg kg^{-1}; 0% nZVI = 0 g kg^{-1}; 0.1% nZVI = 2.5 g kg^{-1}; B0 = 0 g kg^{-1}; B10 = 10 g kg^{-1}; B30 = 30 g kg^{-1}.R.Cd (root cadmium), MDA (malondialdehyde), Sh Cd (shoot cadmium), Rd Cd (reducible cad-mium), S Cd (soil total cadmium), Ox Cd (oxidizable cadmium), AE (acid extractable cadmium), SOD (superoxide dismutase), CAT (catalase), Ch.b (chlorophyll b), Ch.a (chlorophyll a), TC (total chlorophyll), BM (total biomass), Re Cd (residual cadmium), SOC (soil organic carbon), and pH.
